# Early Exposure to Water Turbidity Affects Visual Capacities in Cuttlefish (*Sepia officinalis*)

**DOI:** 10.3389/fphys.2021.622126

**Published:** 2021-02-10

**Authors:** Alice Goerger, Anne-Sophie Darmaillacq, Nadav Shashar, Ludovic Dickel

**Affiliations:** ^1^Normandie Univ., UNICAEN, Ethos (Ethologie Animale et Humaine) UMR 6552, Caen, France; ^2^Department of Life Sciences, Ben Gurion University of the Negev, Eilat, Israel

**Keywords:** optomotor response, linear polarization, vision, cephalopods, development

## Abstract

In La Manche (English Channel) the level of turbidity changes, not only seasonally and daily in seawater but also along the coast. As a consequence, vision in marine species is limited when based only on contrast-intensity. It is hypothesized that polarization sensitivity (PS) may help individuals detect preys and predators in turbid environments. In the cuttlefish, *Sepia officinalis*, to date, all behavioral studies have been conducted on animals reared in clear water. But the cuttlefish sensory system is adapted to a range of turbid environments. Our hypothesis was that rearing cuttlefish in clear water may affect the development of their visual system, and potentially affect their visually guided behaviors. To test this, newly-hatched cuttlefish, from eggs laid by females brought in from the wild, were reared for 1 month under three different conditions: clear water (C group), low turbidity (0.1 g / l of clay, 50–80 NTU, LT group) and high turbidity (0.5 g / l of clay, 300–400 NTU, HT group). The visual capacities of cuttlefish were tested with an optomotor apparatus at 7 days and at 1 month post-hatching. Optomotor responses of juveniles were measured by using three screen patterns (black and white stripes, linearly polarized stripes set at different orientations, and a uniform gray screen). Optomotor responses of juveniles suggest that exposure to turbid water improves the development of their PS when tested in clear water (especially in LT group) but not when tested in turbid water. We suggest that the use of slightly turbid water in rearing systems may improve the development of vision in young cuttlefish with no detrimental effect to their survival rate. Future research will consider water turbidity as a possible factor for the improvement of cuttlefish well-being in artificial rearing systems.

## Introduction

Water turbidity is caused by various mixtures of suspended particles such as sediments, sand/clay (mineral), zooplankton (animal) or algae (plant). These particles absorb and/or scatter the incoming light from the sun. They are also crucial for light and color attenuation in the water column. Light is partially linearly polarized under water. Many factors, such as scattering and the absorption properties of the medium, directionality of the incoming light and the presence of waves on the water surface, can change the orientation of light polarization and induce or reduce polarization [reviewed in Sabbah et al. (2005)]. For example, a little scattering induces polarization but too much scattering reduces the polarization signal (Shashar et al., 2011; Lerner et al., 2012). Turbidity alone and/or combined with other factors may impair the availability and reliability of visual cues for aquatic animals, and thus potentially alter some of their visually guided behaviors.

Visual information is widely used for predator avoidance and/or prey detection in aquatic animals (Luczkovich, 1988; Fuiman and Magurran, 1994; Gall and Fernandez-Juricic, 2010). As a consequence, water turbidity is probably a strong evolutionary constraint for aquatic organisms. In their natural environments, numerous species living in turbid water quite simply reduce their use of vision. For example, the river dolphin living in turbid rivers has eyes of a reduced size (used only as light sensors) as compared to sea dolphins. Some species of river dolphins are blind (Herald et al., 1969; Pilleri, 1979) and rely only on their biosonars to find prey. In other species, the lack of visual information may be balanced by the use of other senses: this is “sensory compensation” (Hartman and Abrahams, 2000). For example, zebrafish reared in clear water rely on visual information in foraging behavior but the ones reared for 2 weeks in turbid water mainly rely on odor information (Suriyampola et al., 2018). In some species, turbidity differences, which are often coupled with spectral changes, affect the developmental plasticity of the visual system. For example, Ehlman et al. (2015) demonstrated a shift from mid-wave-sensitive opsins to long wave-sensitive opsins in guppies (*Poecilia reticulata*) previously reared in turbid water. The visual system has different roles, including but not limited to: detecting brightness, colors, shapes, and motion (Gegenfurtner and Hawken, 1996; Derrington, 2000). In guppies, the change of opsin may increase motion-detecting abilities in this species to balance the loss of color and brightness cues in turbid water. It follows that in order to investigate the effects of turbidity on the development of the visual system, it is appropriate to work with animal models that live in a variety of natural water turbidity conditions and mainly relying on visual information in their basic behaviors.

Cephalopods have keen vision and many of their behaviors are guided by visual information. There is a great plasticity of their visual capacities and subsequent behaviors depend on experience during the early life stages (Huffard, 2013; Darmaillacq et al., 2017; Marini et al., 2017; Mather and Dickel, 2017; Villanueva et al., 2017). Cephalopods are colorblind (Marshall and Messenger, 1996; Mäthger et al., 2006) but most species have polarization sensitivity (PS), i.e., they can detect the *e*-vector orientation and the degree (percent) of linear polarization of the incoming light. Since no cephalopod is known to be sensitive to the circular polarization component of light, we refer here only to linear polarization without specifying this further. Cuttlefish probably show the finest *e*-vector angle discrimination of all cephalopods (Temple et al., 2012) and are consequently a particularly valuable model for the study of PS. In cuttlefish, PS is potentially involved in various functions such as communication (Shashar et al., 1996; Boal et al., 2004), orientation (Cartron et al., 2012), prey detection (Shashar et al., 1998, 2000) and predator detection (Cartron et al., 2013b,c). Cartron et al. (2013c) demonstrated that PS increases visual capacities in a turbid environment in cuttlefish (*S. officinalis*, *S. pharaonis*, and *S. prashadi*).

A powerful and simple way to study the visual capacities of animals is to measure their optomotor response (OMR) to different visual stimuli (mostly a screen with contrasted patterns rotating around the animal, McCann and MacGinitie, 1965; Groeger et al., 2005; Rinner et al., 2005). When presented with a moving stimulus an individual exhibits unconditioned movements of its eyes, head or whole body following the direction of the moving stimulus (Darmaillacq and Shashar, 2008). OMR can be used to examine sensitivity to contrasts, spectral sensitivity or PS (Darmaillacq and Shashar, 2008). Cartron et al. (2013a) used OMR to show differences of visual capacities based on intensity and polarization contrasts in young cuttlefish previously reared in clear water (from hatching to 30 days of age). Sensitivity to contrast was high from the time of hatching. By contrast, only 20% of individuals responded to polarized stripes patterns at the hatchling stage but all responded to the polarized signal at 1 month. This can be linked, at least partially, to the delay between hatching and first prey catching (Dickel et al., 1997).

*Sepia officinalis*, a common species, breeds, hatches, and develops in the turbid water of La Manche (English Channel). Up to now, developmental studies on cuttlefish vision (including our own) have always been conducted on animals previously reared in clear water. The present study will investigate the development of visual capacities in young cuttlefish previously reared in different water turbidities. We hypothesize that (1) In turbid water, information based on intensity contrast will be less well perceived than that based on PS. (2) Cuttlefish reared in turbid water will develop PS faster and will consequently display better vision in turbid water than those reared in clear water. These results could provide valuable information about the water quality standards to be used in cuttlefish rearing systems under laboratory conditions according to current European regulations (Directive 2010/63/EU).

## Methods

### Animals

Eggs from the wild were collected from several egg batches in Luc-sur-Mer/Villers-sur-Mer and Arcachon vicinities in France (Normandy and Gironde, respectively). They were separated and put randomly in baskets in shallow tanks at the Centre de Recherches en Environnement Côtier (CREC, Luc sur Mer, France). The system is an open system with a flow rate of about one liter/min to avoid any recycling of turbidity. All tanks were then supplied with running oxygenated clear sea water at 19 ± 1°C. After hatching, cuttlefish were reared for 1 month under three conditions: clear water (C), slightly turbid water (LT), and highly turbid water (HT) in tanks (40 cm × 60 cm × 32 cm) providing an enriched habitat (artificial plants, stones, and shells). Each tank contained a maximum of 30 animals at the same time. All tanks were cleaned daily to avoid the proliferation of bacteria and waste matter. Cleaning was done when the water was clear to avoid damaging the animals. The procedure was the same for all groups. Then, one liter of seawater, without clay (C) or with clay (LT and HT), was added each day. The amount of clay was calculated to obtain a turbidity of 0.1 g/l (50–80 NTU) in the LT and 0.5 g/l (300–400 NTU) in the HT. Turbidity of the water of each tank was measured using a turbidimeter (Turbidimeter 2016LM). Animals were fed daily *ad libidum* with live shrimps (*Crangon crangon*) just after the daily turbidity measurement. Sixty-two cuttlefish were tested at 7 days post hatching (*n* = 20 for C and HT group and *n* = 22 for LT group) and 30 other cuttlefish at 30 days of age post hatching (*n* = 10 in each group). These ages were chosen in accordance with Cartron et al. (2013a) that showed that the visual system critically develops during the month of life. Animal maintenance and experimentation were in compliance with the Directive 2010/63/EU on the protection of animals used for scientific purposes, and following the recommendations of the 3Rs (Fiorito et al., 2014).

### Optomotor Apparatus and Behavioral Tests

**FIGURE 1:**
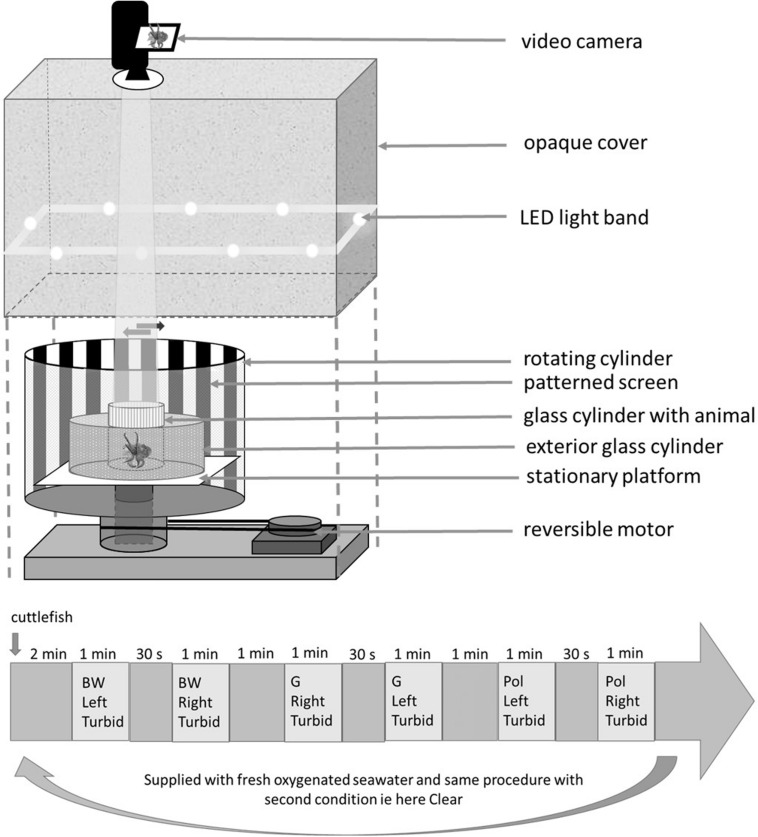
Optomotor apparatus and an example of a procedure for which the order was randomized. The apparatus consisted of a cylinder showing three patterns (BW, black and white; G, gray; Pol, polarized stripes.) and rotated by a reversible motor placed on the inner wall of the apparatus. Two glass cylinders, one holding the animal and the other with more or less turbid water, were placed on a stationary platform in the center of the apparatus. In the procedure, the external cylinder was supplied with turbid seawater (condition 1) and for condition 2 the same experiment was done with clear seawater. Between the two conditions the cylinder holding the animal was supplied with fresh oxygenated seawater.

The optomotor system was described in detail in Darmaillacq and Shashar (2008). In short, the method is based on evoking conditioned OMR (eye or body movements) of cuttlefish with the rotation of contrasting stripes. When rotated stripes are perceived, the cuttlefish will follow the direction of the pattern movements with its eyes or its whole body in order to stabilize the moving visual field. Briefly, the optomotor apparatus consists of a cylinder (40 cm diameter) rotated by a controllable, reversible motor. The patterned screen was placed on the interior wall of the cylinder and a light diffuser was put on the exterior wall. In the center of the apparatus two glass cylinders (one holding the animal, 12 cm diameter and the other containing either clear or turbid water, 24 cm diameter) were placed on a stationary platform. Adding another compartment to the Darmaillacq and Shashar (2008) OMR device allowed us to test the visual ability of young cuttlefish in both clear and turbid water. To avoid any experimenter disturbance, the entire apparatus was covered with an opaque curtain with a single hole for a video camera just above the glass cylinder containing the animal. A LED band placed around the tank with light diffusers provided uniform and stable lighting of the pattern during the experiment. We tested two patterns with 10 mm wide stripes: black and white alternating stripes (BW) and polarized stripes (Pol). The latter consisted of alternating stripes with different orientations of linear polarization (see methods in Cartron et al., 2013a): 0°, 45°, 90°, and 135° (sheet #318, Frank Woolley & Co.). As a control, cuttlefish were tested with a uniform sheet of gray paper (G) (Figure 1). Preliminary tests showed the same OMR for cuttlefish when using black and white alternating stripes or black, white and two shades of gray alternating stripes used in Cartron et al. (2013a). Thus it seems that the complexity of the pattern did not interfere with the visual ability of cuttlefish. To check whether the use of two glass cylinders in the apparatus could interfere with stripe detection a video camera was put inside the apparatus instead of the cuttlefish. The contrast between the stripes was measured using ImageJ software and Michelson formula for both stripes (BW and Pol). In clear water there was no difference between contrasts measured through a single or double cylinder (respectively, 43 and 37% contrast difference between the polarized stripes).

Following Cartron et al. (2013a), each cuttlefish was gently moved from its home tank to the experimental cylinder. It was allowed 2 min to calm down before the beginning of the experiment. Following preliminary tests, one speed 30°/s was used for each pattern turning in two directions (clockwise and counter-clockwise). Each cuttlefish was tested twice: first surrounded by clear water and then by turbid water (0.1 g/l of clay mixed with water i.e., low turbidity), in a random order. For both conditions the cuttlefish was submitted to six trials (three patterns × two directions × one speed) for a maximum duration of 15 min each. Between tests in clear and turbid water, the cuttlefish was supplied with new, oxygenated water from its home tank (this water was taken before the turbid event in order to keep the water in the experimental cylinder clear for all groups). Patterns (BW, Pol, and G), directions (clockwise and counter-clockwise) and conditions (turbid or not) were chosen for each session (see the Figure 1 for a combination example). The interval between two directions was 30 s. There were 1-min intervals between two patterns and 2-min intervals between two conditions. Responses were considered as positive when a cuttlefish followed the patterns over at least 180° in both directions and did not show any response to the control sheet (G) i.e., as in Cartron et al. (2013a) (Figure 1). It must be specified that we were stricter in scoring than in previous studies. A response was only considered to be positive with at least a 180° rotation of the tested animal instead of the 90° cutoff in Groeger et al. (2005) and Darmaillacq and Shashar (2008). Furthermore, in the present study a response was only considered to be positive when the animal responded to both rotational directions, single responses being ignored (for an example of a positive response see the video in Supplementary Material).

### Statistical Analyses

Data were analyzed using R version 3.5.2. Non-parametric McNemar test for paired data with continuity correction was used to compare responses between the two experimental conditions (turbid or not) as well as the responses for the two patterns. For comparison between the three groups a Fisher–Freeman–Halton test (Fisher’s exact test for count data with simulated p-value based on 10^5^ replicates) was used in addition to a *post-hoc* pairwise Fisher’s test with Bonferroni corrections. Comparisons between ages were determined with Fisher’s test. Cutoff for significance was decided as *p* < 0.05.

### Ethics Statement

This research followed the guidance by Directive 2010/63/EU, and French regulations regarding the use of animals for experimental procedures, and was approved by the Regional Ethical Committee Cenomexa [Project agreement number: APAFIS 2019100316587299 _V2 (#20662)]. The experiment was designed to decrease animal distress by minimizing the number of animal.

## Results

The OMR was recorded in three groups of cuttlefish: a control group reared in constantly clear seawater (C group) and two groups reared in turbid sea water; one group with low turbidity (LT group) and one with high turbidity (HT group). All animals were tested at two ages: 7 days and 1 month post hatching. To assess the effect of turbidity on the development of luminance and PS, we used two patterns, respectively, a BW pattern (black and white stripes) and a Pol pattern (polarized stripes). A uniform gray sheet served as a control pattern (no response expected). Each pattern was tested in two experimental conditions: clear water and turbid water.

At 1 month, the LT group had a somewhat higher survival rate (C group survival = 87.5%; LT group survival = 95%; HT group survival = 85%). Mean cuttlefish size (Dorsal Mantle Length, DML) was slightly greater in the C group at both ages (at 7 days C DML = 10.9 ± 1.1 mm, *n*_*C7*_ = 20; LT DML = 10.8 ± 1.0 mm, *n*_*LT7*_ = 22; HT DML = 10.6 ± 1.4 mm, *n*_*HT7*_ = 20; at 1 month C DML = 18.4 ± 1.5 mm, *n*_*C30*_ = 10; LT DML = 17.6 ± 1.9 mm, *n*_*LT30*_ = 10; HT DML = 17.9 ± 2.2 mm, *n*_*HT30*_ = 10).

**FIGURE 2:**
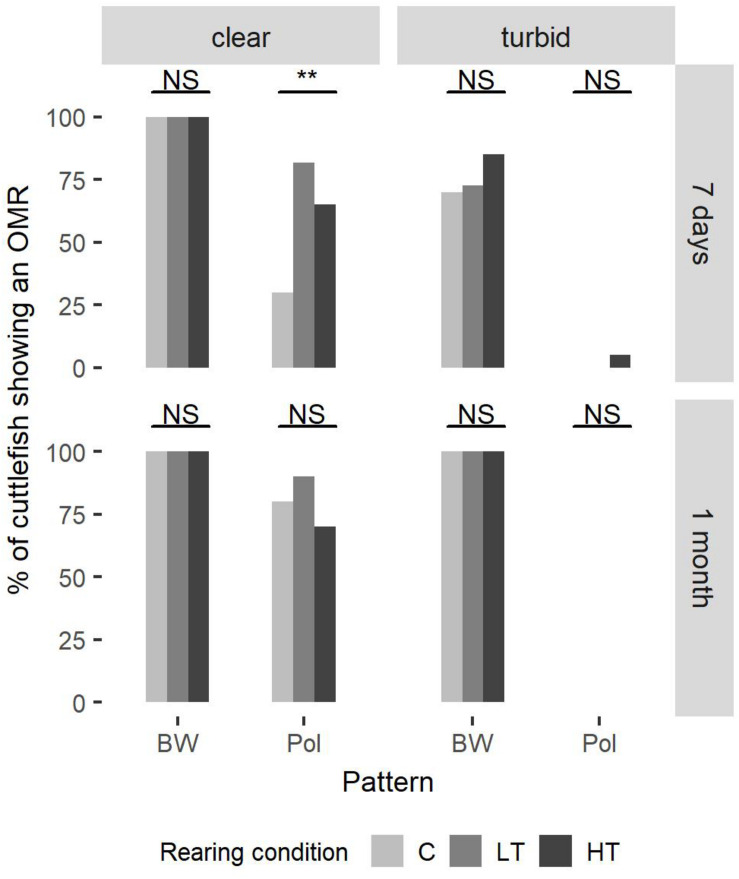
The percentage of cuttlefish showing an optomotor response under three rearing conditions. At 7 days post hatching, 20 cuttlefish were tested in groups C and HT and 22 cuttlefish in group LT. At 1 month post hatching, 10 cuttlefish were tested in each group. Fisher–Freeman–Halton test (Fisher’s exact test for count data with simulated p-value based on 10^5^ replicates) was used to compare the three groups, with the addition of a post-hoc pairwise Fisher’s test with Bonferroni corrections. At 7 days in clear water there is significant polarization sensitivity (PS) difference between the groups LT (80%) and C (30%) (*P* = 0.0044).

In clear water all animals (both ages) showed sensitivity to light intensity (BW) (Fisher-Freeman–Halton test, 7 days-clear water, *P* = 1.00, *n*_*C*_ = *n*_*HT*_ = 20, and *n*_*LT*_ = 22; 1 month-clear water, *P* = 1.00, *n*_*C*_ = *n*_*LT*_ = *n*_*HT*_ = 10) (Figure 2).

However, in clear water PS improved with development, especially in group C (Fisher test; *P* = 0.019, *n*_*C7*_ = 20, and *n*_*C30*_ = 10) (data not shown). At 7 days in clear water the response rate was higher (70–80%) in HT and LT groups (70 and 80%, respectively) with significant PS difference between the group LT (80%) and the group C (30%) (*P* = 0.0044, *n*_*LT*_ = 22, and *n*_*C*_ = 20) (Figure 2).

At 7 days and in clear water, the groups C and HT had a higher response rate for the intensity pattern than for the polarized pattern (McNemar test; C, χ^2^ = 12.07, df = 1, *P* < 0.001; HT, χ^2^ = 5.14, df = 1, *P* = 0.023, *n*_*C*_ = *n*_*HT*_ = 20) (data not shown). At 1 month, this difference disappeared (McNemar test; C, χ^2^ = 0.5, df = 1, *P* = 0.48; HT, χ^2^ = 1.33, df = 1, *P* = 0.25, *n*_*C*_ = *n*_*HT*_ = 10) (Figure 2).

In turbid conditions sensitivity to intensity (BW) increased with development (McNemar test; C, χ^2^ = NaN, df = 1, *P* = 1.00, *n*_*C7*_ = 20, and *n*_*C30*_ = 10; LT, χ^2^ = NaN, df = 1, *P* = 1.00, *n*_*LT7*_ = 22, and *n*_*LT30*_ = 10; HT, χ^2^ = NaN, df = 1, *P* = 1.00, *n*_*HT7*_ = 20, and *n*_*HT30*_ = 10) (Figure 2). Indeed, under turbid conditions only 70% (group C) to 85% (group HT) of the 1-week old cuttlefish showed a response. The groups C and LT had lower response rate in these experimental conditions than in clear water conditions (McNemar test; C, χ^2^ = 4.17, df = 1, *P* = 0.041, *n*_*C*_ = 20; LT, χ^2^ = 4.17, df = 1, *P* = 0.041, *n*_*LT*_ = 22) (Figure 2).

Polarization sensitivity in turbid water was significantly lower than in clear water (McNemar test; C-7 days, χ^2^ = 4.17, df = 1, *P* = 0.042, *n*_*C7*_ = 20; LT-7 days, χ^2^ = 16.06, df = 1, *P* < 0.001, *n*_*LT7*_ = 22; HT-7 days, χ^2^ = 8.64, df = 1, *P* = 0.0033, *n*_*HT7*_ = 20; C-1 month, χ^2^ = 6.13, df = 1, *P* = 0.013, *n*_*C30*_ = 10; LT-1 month, χ^2^ = 7.11, df = 1, *P* = 0.0077, *n*_*LT30*_ = 10; HT-1 month, χ^2^ = 5.14, df = 1, *P* = 0.023, *n*_*HT30*_ = 10) (Figure 2). In fact, in turbid water, at 7 days, no animal showed any OMR, with the exception of one cuttlefish from HT group.

On the other hand, in turbid water the response rate for the intensity pattern was always higher than for the polarized pattern for all groups and both ages (McNemar test; C-7 days, χ^2^ = 12.07, df = 1, *P* < 0.001, *n*_*C7*_ = 20; LT-7 days, χ^2^ = 14.06, df = 1, *P* < 0.001, *n*_*LT7*_ = 22; HT-7 days, χ^2^ = 12.5, df = 1, *P* < 0.001, *n*_*HT7*_ = 20; C-1 month, χ^2^ = 8,1, df = 1, *P* = 0.0044, *n*_*C30*_ = 10; LT-1 month, χ^2^ = 8,1, df = 1, *P* = 0.0044, *n*_*LT30*_ = 10; HT-1 month, χ^2^ = 8,1, df = 1, *P* = 0.0044, *n*_*HT30*_ = 10) (Figure 2).

## Discussion

The aim of this study was to investigate effects on their visual capacities when rearing young cuttlefish in turbid or clear water. Whatever the water turbidity in the rearing system, no abnormal behavior (abnormal swimming, difficulty to catch prey, skin discoloration or damage) was observed in any animal. However, the survival rate of juveniles was somewhat better in cuttlefish reared with low turbidity than that of animals kept in clear water.

When tested in clear water, PS improved with development. This is in accordance with the results of Cartron et al. (2013a). However, PS was significantly higher at 7 days in LT group (Figure 2). One can hypothesize that exposure to turbid water during early development can improve the development of PS in cuttlefish. Since young cuttlefish mainly prey upon mysids, which are transparent, the earlier their PS development, the higher their predation success. Domingues et al. (2004) showed that when fed transparent shrimps, rather than fish, young cuttlefish show higher growth and survival rates under laboratory conditions. As a consequence, constant use of clear water in a cuttlefish-rearing system may reduce the need of PS improvement to catch preys and may thus negatively impact PS development of juveniles, and hence their fitness. In addition, low turbidity may provide optimized conditions for reducing incoming light from the rearing system, thus reducing individual stress. Low turbidity may also facilitate concealment and mutual avoidance between individuals.

When tested in turbid water, sensitivity to light intensity (BW stripes) improved with development in all groups. However, the higher the turbidity in the rearing system the better the light-intensity sensitivity at 7 days. These results confirm for the first time the link between turbid-water rearing conditions and visual capacity improvement in young cuttlefish. However, in nearly all cases, there was no response to the polarized signal when tested in turbid waters. PS, more than light-intensity sensitivity, seems to be specifically limited by the turbidity of the water in juvenile cuttlefish. This is in contradiction with a paper by Cartron et al. (2013b, c), according to which PS improves vision in turbid water in 5-month old cuttlefish. However, in the case in question turbid water was obtained by mixing fine sand (Cartron et al., 2013a,b) whereas we used clay in the present study. In water, partially polarized light is subjected to scattering and absorption by content in suspension (Lerner, 2014). These effects on PS were size- and concentration-specific but not shape-specific [model with spherical particles hypothesis from Lerner et al. (2012) succeeded in explaining measured data in the field in Lerner et al. (2011)]. Fine sand particles and clay particles vary in size (above 50 and 20 μm, respectively). Mie particles (spherical particles with a size between 2 and 20 microns) depolarize the light (and reduce the polarization contrast) whereas geometric particles (size above 100 microns) increase partial polarization (Lerner, 2014). Therefore, different types of sediment creating turbidity may have a strong impact on the transmission of polarized signals. Indeed, Bainbridge and Waterman (1958) showed that mysid crustacean orientation with polarized stimuli improved with water turbidity, and suggested that turbidity created an additional intensity signal related to the polarization of the incoming light. They further speculated that this intensity signal may have overridden the original polarization signal and influenced the shrimps’ behavior.

Our results are unexpected, suggesting that future studies could examine their relevance to the real-life situation of cuttlefish. On the other hand, turbid water may offer an attractive environment for an ambush-predator like cuttlefish. It is interesting to note that fishermen usually collect mysids (cuttlefish preys) in turbid areas (Dickel personal observations from different locations in Luc sur Mer and Galveston, TX vicinities). In the present work, the conditions of turbidity (episodic events) in the rearing tank were maybe less frequent (once a day) than those experienced by wild juveniles in the field. As a consequence, more animals may develop PS in their natural environment. Turbid water in the field comprises a mixture of different particles such as fine sand, clay, other minerals and plankton and may well allow the cuttlefish to use PS to visually discriminate between transparent prey, its surroundings (Sabbah and Shashar, 2006; Johnsen et al., 2011) and predators (Cartron et al., 2013b,c). Future studies should explore the effect of water turbidity on PS when created with two components (algae and clay for example) as well as the single effect of each component. As an example, Nieman et al. (2018) showed that visual detection thresholds of two fishes (*Notropis atherinoides* and *Sander vitreus*) were more altered by algal turbidity compared to sedimentary turbidity. This study also demonstrated that the effect of combination treatment (algal and sedimentary turbidity) not only slightly decreased the amount of light (11%) when compared to the separate component (algal turbidity reduced it by 42% and sedimentary turbidity by 35%) but also green-shifted the light as with the algae treatment. As a result it would be difficult to predict the water turbidity effect on vision based only on the water turbidity concentration. However, direct measurements of stripe contrasts in variegated turbidities (using algae, sand, clay or combined ingredients) would provide valuable information. To state the obvious, cuttlefish possess a range of senses such as hearing (Komak et al., 2005) and smell (Boal and Golden, 1999) which may be used in parallel or alternately when one of the other senses is less efficient.

Multiple studies show that the environmental enrichment of a home tank improves the visual abilities (Cartron, 2012) or cognitive and memory skills (Dickel et al., 2000) of the cuttlefish. Environmental enrichment consists of adding stones, sand, shelters, and artificial plants but there is no study on how water turbidity could have an effect on cuttlefish welfare and fitness. The present study demonstrated that rearing cuttlefish in clear water could alter PS development when compared to low turbidity conditions. In addition low turbidity may reduce incoming light from the rearing system, thus reducing individual stress. Low turbidity may also facilitate concealment and mutual avoidance. It should be noted that a study by O’Brien et al. (2016) showed no difference in either predation or camouflage behaviors between cuttlefish reared in the wild (until 2 weeks before hatching) and those reared in clear water in laboratory conditions. However, there was an exception with a uniform pattern, when laboratory-reared cuttlefish produced better camouflage on a uniform background than those from the field). But in the latter study, the wild cuttlefish spent only the first part of their embryonic development in their natural environment, which may not have been sufficient to elicit behavior plasticity. The present study suggests that creating slight turbidity, possibly as a temporary change of the visual context, may improve rearing conditions for young cuttlefish. Further study is necessary to assess the long-term effects of rearing-system water turbidity on the sensory skills and behaviors of juvenile and adult cuttlefish. Such a study would first also help to determine the maximum turbidity level the cuttlefish can tolerate (to maximize survival). Second, it has to be checked that the turbidity of the water brings an actual increase of cuttlefish survival (assessed by daily measurement of cuttlefish size, food consumption, survival rate at each age), which would counterbalance the cost for extra maintenance (more cleaning due to the sediment in the pipes, tanks, etc.).

## Data Availability Statement

The raw data supporting the conclusions of this article will be made available by the authors, without undue reservation.

## Author Contributions

AG and LD: conception and design of the experiment. AG: data acquisition and data analysis. All authors have taken active part in data interpretation, discussions, and preparation of the manuscript.

## Conflict of Interest

The authors declare that the research was conducted in the absence of any commercial or financial relationships that could be construed as a potential conflict of interest.
